# An Investigation of the Properties of Expanded Polystyrene Concrete with Fibers Based on an Orthogonal Experimental Design

**DOI:** 10.3390/ma15031228

**Published:** 2022-02-07

**Authors:** Yi Sun, Chenxi Li, Junjie You, Changming Bu, Linwen Yu, Zhitao Yan, Xinpeng Liu, Yi Zhang, Xianrui Chen

**Affiliations:** 1 School of Civil Engineering and Architecture, Chongqing University of Science & Technology, Chongqing 401331, China; sunyi@cqust.edu.cn (Y.S.); lichenxi@cqust.edu.cn (C.L.); yzt@cqust.edu.cn (Z.Y.); liu_simple@cqust.edu.cn (X.L.); 2 Chongqing Key Laboratory of Energy Engineering Mechanics & Disaster Prevention and Mitigation, Chongqing 401331, China; 3 School of Civil Engineering, Southwest Jiaotong University, Chengdu 610031, China; 4 College of Materials Science and Engineering, Chongqing University, Chongqing 400044, China; 5 Chongqing Construction Residential Engineering Co., Ltd., Chongqing 400015, China; kzb@cqzj.com.cn; 6 Chongqing Tidy Green New Material Co., Ltd., Chongqing 401221, China; 2012026@cqust.edu.cn

**Keywords:** EPS concrete, fiber-reinforced concrete, orthogonal experimental design, mechanical properties, microstructures

## Abstract

Expanded polystyrene (EPS) concrete is commonly used as the core material of commercial sandwich panels (CSPs). It is environmentally friendly and lightweight but has poor strength. Adding fibers can improve the microstructure of EPS concrete and reduce the weakening effect of EPS beads on the mechanical properties of concrete. An orthogonal experimental design (OED) was used in this paper to analyze the influence of length and content of polypropylene fiber (PF), glass fiber (GF), and carbon fiber (CF) on the physical and mechanical properties and micromorphology of EPS concrete. Among them, CFs have the most apparent impact on concrete and produce the most significant improvements in all properties. According to the requirements of the flexural performance of CSPs, the splitting tensile strength was taken as the optimization index, and the predicted optimal combination (OC) of EPS concrete with fibers was selected. The variations in the material properties, mechanical properties, and microstructure with age were analyzed. The results show that with increasing age, the dry density, compressive strength, and splitting tensile strength of concrete are markedly improved relative to those of the CSP core material and the control case (CC), and even the degree of hydration is improved.

## 1. Introduction

Expanded polystyrene (EPS) beads are byproducts of petroleum engineering and have the advantages of facility acquisition low densities, economical prices, low water absorption, and great thermal insulation performance. However, they still have the following disadvantages: they are nondegradable, difficult to recycle and treat, and they have poor mechanical strength. EPS beads can be used for the partial replacement of raw aggregates and can be made into EPS concrete, which not only utilizes industrial waste but also saves the usage of aggregates and reduces the density and weight of concrete. It is worth noting that EPS concrete is fairly environmentally friendly.

Owing to the low density of EPS beads, which is generally lower than 20 kg/m^3^, the density of concrete can be sharply reduced by adding EPS beads. As the proportion of EPS increases, the density, compressive strength, splitting tensile strength, and elastic modulus of concrete present decreasing trends [1,2,3,4,5]. Moreover, the size of the EPS beads also has an obvious effect on the material properties [6] under the same density of EPS concrete. As the size decreases, the compressive strength performance of concrete and the binding force between cement and EPS beads are both enhanced [1,7].

Fly ash (FA) is used as supplementary cementitious material, and it improves the water absorption rate [8] and reduces the dry shrinkage of concrete. Silica fume (SF) is an industrial byproduct that is suitable for filling cement pores [9] due to its small diameter, and by this way, the durability could be improved [10]. In addition, the mechanical properties of concrete can be properly improved by the gel that is formed by rehydration with cement hydration products and SF. Similarly, the addition of SF not only improves the durability of the concrete [11] but also enhances the compressive strength and reduces the porosity. In particular, it can tightly connect the interfacial transition zone (ITZ) between EPS beads and the interface of concrete, which improves the segregation of materials [12].

EPS beads have minimal mechanical strength. Thus, the strength of EPS concrete is mainly determined by the strength of concrete without EPS beads and its microstructure. The ordinary method for enhancing the mechanical strength of EPS concrete without affecting its low density and lightweight design is to add fibers. Fibers have a significant enhancement effect on the mechanical properties of concrete [13,14,15,16]. Typically, PF [17,18,19], GF [20,21], and CF [22,23] are commonly used as reinforcing materials to enhance the various properties of concrete. The addition of fibers has improved the anti-shrinkage, splitting tensile strength, compressive strength, and flexural properties of EPS concrete to varying degrees. As the proportion of fibers increases, the compressive strength also increases. However, when the proportion of fibers exceeds 1% [24], the compressive strength exhibits a downward trend with an increase in the proportion of fibers [25,26,27,28,29]. For EPS concrete, the peak value of the compressive strength is affected by the different proportions of fibers, but the overall variation trend is the same [19]. Correspondingly, fibers can upgrade the microstructure of concrete to a certain extent and improve the mechanical properties accordingly. Therefore, it is a major tendency to use fibers to compensate for the weakening effect of EPS on concrete.

In summary, the mechanical properties of EPS concrete are severely weakened due to the poor mechanical properties of the EPS beads added to concrete. Nevertheless, recent research on fiber-modified EPS concrete is relatively limited, especially research on the various properties at different ages. Therefore, it is necessary to investigate the influence of various factors of fibers on the material and mechanical properties of concrete.

In this paper, the orthogonal test method is mainly used to investigate the influence of the three factors of fibers, which are the type, length, and proportion, on the various properties of EPS concrete. The physical properties, mechanical properties, and micromorphology of the specimens are investigated. On this basis, the application of sandwich panels is taken as an example, and the mechanical properties and microscopic characteristics of EPS concrete at various ages are discussed according to the requirements of improving the splitting tensile strength.

## 2. Materials and Methods

### 2.1. Materials

In this paper, ordinary Portland cement (OPC) (manufactured by Yangchun Cement Co., Ltd. from Weifang, China) according to the requirements of P. O. 42.5 in GB175-2007 [30] and Type I in ASTM C150-20 [31] was used. The chemical composition and performance indicators are shown in Table 1. SF with a particle size of 0.1~0.3 μm was supplied, which met the requirements of GB/T 27690-2011 [32] and ASTM C1240-20 [33] The chemical composition is shown in Table 2. Natural river sands (NRSs) were used as fine aggregates and tested by the screening method and moisture proportion measurement method specified in GB/T 14684-2011 [34]. The results are shown in Table 3. The NRSs belonged to natural sand grade type I with a fineness modulus of 1.73, moisture content of 9.61%, and a bulk density of 1664 kg/m^3^. A polycarboxylate-based superplasticizer (SP) (S04B type) was adopted.

**Figure 1:**
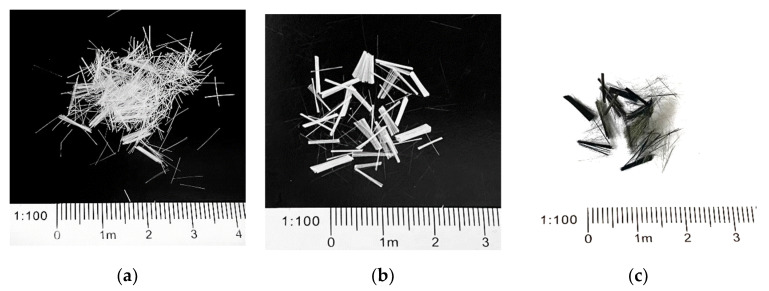
Macroscopic morphology of the fibers: (**a**) PF; (**b**) GF; (**c**) CF.

EPS beads with an apparent density of 25.1 kg/m^3^ and a bulk density of 11.31 kg/m^3^ were adopted and tested under the screening method and bulk density measurement method specified in GB/T 17431-2010 [35] and ASTM C136-19 [36]. The fibers used in this article included PF, GF, and CF, which had lengths of 6 mm, 9 mm, and 12 mm, respectively. The macrostructure and main physical performance of the fibers are separately shown in Figure 1 and Table 4.

### 2.2. Mix Design

An orthogonal experimental design (OED) was adopted to determine the optimal indexes of the fibers, with three factors and three levels, as shown in Table 5. Thereinto, the three factors are fiber type, fiber length, and fiber dosage, and the three levels correspond to corresponding factors, respectively.

EPS concrete without fiber was added as the control case (CC) based on the OED. The naming rules are as follows: the first letter is fiber type, the first two digits are fiber length, and the last two digits are fiber content. For instance, P0605 means choosing PF with a length of 6 mm and a dosage of 0.5%. The mixed number of other contents such as cement, SF, water, NRS, SP [37], and EPS, was decided by the previous test study. The mix design is shown in Table 6. The concrete mixing process is as follows. First of all, the cement, silica fume (SF), fly ash (FA), and river sand are dry mixed for 1 min. After the solid mixture is mixed evenly, the liquid superplasticizer (SP) and water are added stirring for 5 min; then the expanded polystyrene (EPS) beads are added to the mixture continue stirring for 2 min, forming a stable and uniform fresh concrete mixture. Finally, adding various types of fibers are stirred for another 2 min, and loaded into the concrete standard mold.

### 2.3. Experimental Methods

Slump and dry density tests were performed according to GB/T 50080-2016 [38], and the water absorption test was related to JG/T 266-2011 [39]. To prevent the EPS particles in the concrete from being in a molten state due to high temperatures, a thermostatic drying oven at 60 °C was used. The dry shrinkage test was measured by a concrete shrinkage expansion machine HSP 540 (manufactured by ZKLD from Beijing, China), according to GB/T 50082-2009 [40], for the ages of 1 day, 3 days, 7 days, 14 days, 28 days, 45 days, 60 days, 90 days and 120 days, and the size of the specimen was 100 mm × 100 mm × 515 mm. In addition, the ultrasonic pulse velocity (UPV) test was carried out using a ZBL-U520 nonmetallic ultrasonic detector according to ASTM C597-16 [41] and ACI 228.1R-03 [42]. The compressive strength, splitting tensile strength, uniaxial compressive strength, and static elastic modulus tests were by GB/T 50081-2019 [43].

A KYKY-EM6200 (manufactured by KYKY from Beijing, China) scanning electron microscope (SEM) and SmartLab-9 (manufactured by Rigaku from Japan) diffractometer for X-ray diffraction (XRD) were used to analyze the microstructure and various diffraction phases of concrete. The scanning voltage, electric current, speed, and scanning 2θ range were 45 kV, 200 mA, 12°/min (step size was 0.02°), and 5–60°, respectively.

## 3. Results and Discussion

The test results of the material and the mechanical properties with the curing age of 28 days of concrete are shown in Table 7. 

### 3.1. Properties of Fresh Concrete

Table 6 shows that the slump ranges from 1 mm to 115 mm. The minimum value of C0615 and C0910 was 1, the maximum value of P0605 was 115, and the value of CC was 196 mm.

**Figure 2:**
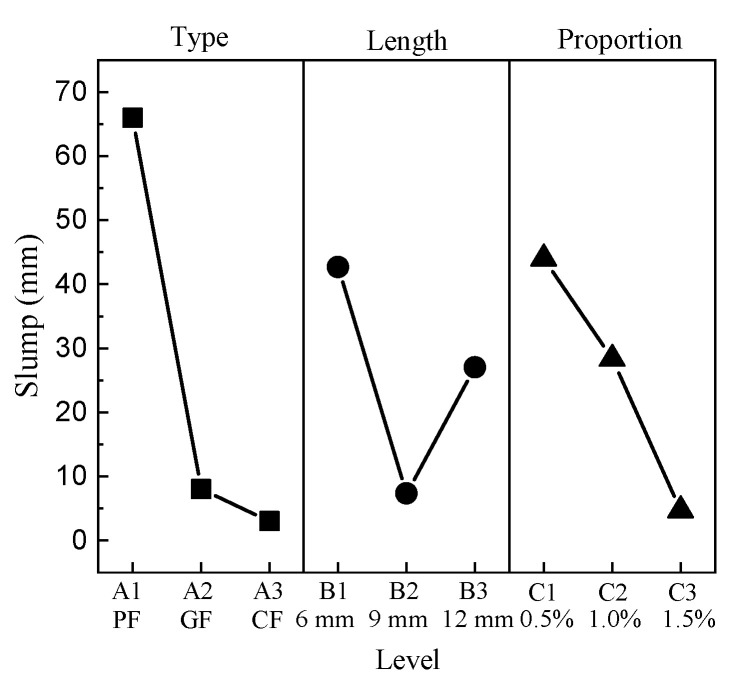
Mean slump response.

The results show that the addition of fibers can greatly enhance the consistency and reduce the flowability of concrete, resulting in a decrease in workability. The mean response analysis of the slump values is shown in Figure 2.

For the type of fiber factor, the maximum is level 1, and the minimum is level 3. For the length factor, the minimum value appears at level 2, and the maximum value appears at level 1. The slump has strong negative correlations with the proportion of fibers; namely, the minimum value is at level 3 and the maximum value is at level 1.

### 3.2. Dry Density

**Figure 3:**
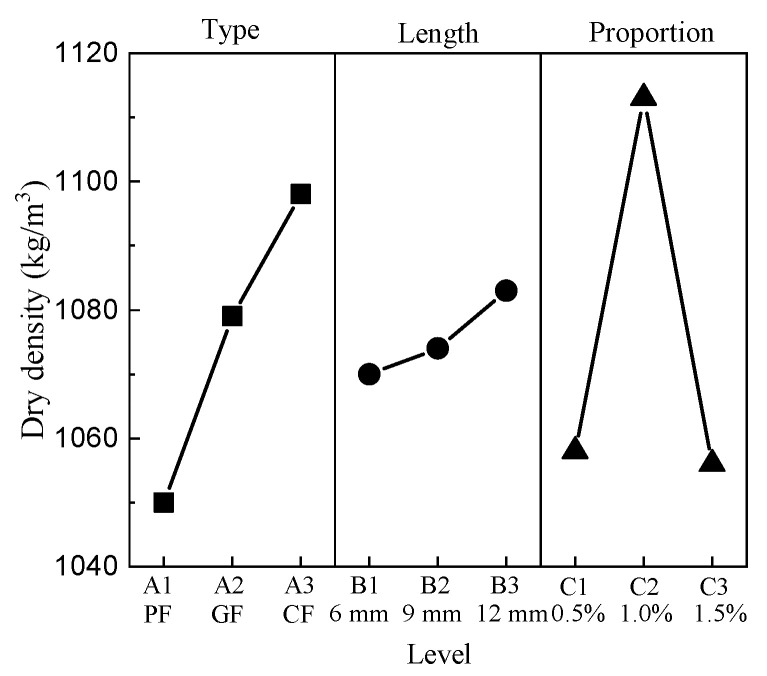
Mean dry density response.

The dry density ranges from 986.42 kg/m^3^ to 1151.2 kg/m^3^. P0915 has the minimum dry density value, P1210 has the maximum dry density value, and the dry density of CC is 1036.6 kg/m^3^. The results show that the addition of fibers can influence the dry density to some degree. The mean response analysis of the dry density is shown in Figure 3.

For the type of fiber factor, the minimum is level 1, and the maximum is level 3. The dry density has significant positive correlations with the lengths of fibers; namely, the minimum value is at level 1 and the maximum value is at level 3. For the proportion factor, the minimum value appears at level 3, and the maximum value appears at level 2.

### 3.3. Water Absorption

**Figure 4:**
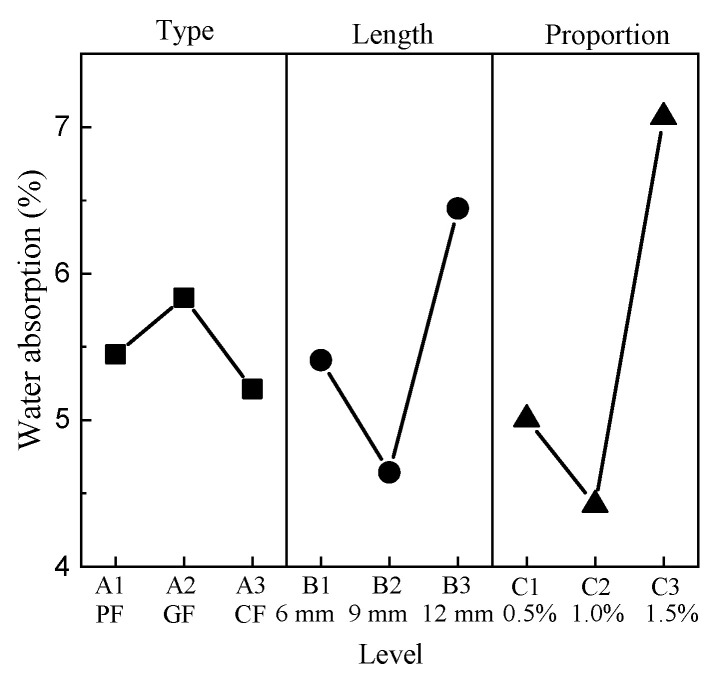
Mean water absorption response.

The water absorption rate ranges from 3.5% to 9.5%. G0905 has the minimum value, G1215 has the maximum value, and the water absorption of CC is 5.5%. The results show that the addition of fibers has a certain effect on water absorption. The mean response analysis of water absorption is shown in Figure 4.

The minimum value of the type factor appears at level 3, and the maximum value appears at level 2. For the length factor, the minimum value appears at level 2, and the maximum value appears at level 3. The minimum value of the proportion factor appears at level 2, and the maximum value appears at level 3.

### 3.4. Ultrasonic Pulse Velocity

**Figure 5:**
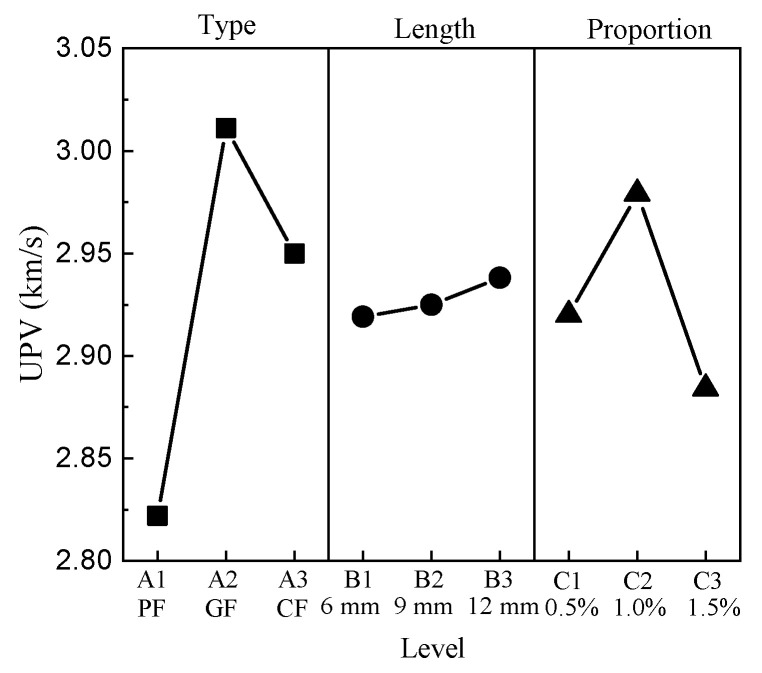
Mean UPV response.

The UPV ranges from 2.74 km/s to 3.02 km/s. P0515 has the minimum value, G0905 and G0910 have the maximum values, and the UPV of CC is 2.86 km/s. The results show that the addition of fibers has an influence on the UPV to a certain extent. The mean response analysis of the UPV is shown in Figure 5.

For the type of fiber, the minimum value of the type factor appears at level 1, and the maximum value appears at level 2. The UPV is positively correlated with the lengths of fibers, where the minimum value is at level 1 and the maximum value is at level 3. The minimum value of the proportion factor appears at level 3, and the maximum value appears at level 2.

### 3.5. Dry Shrinkage

**Figure 6:**
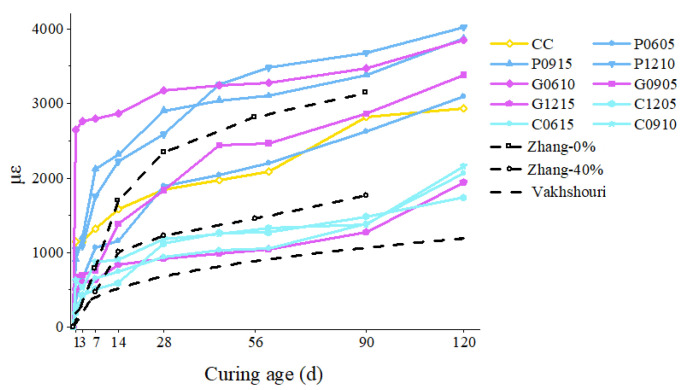
The trend of the dry shrinkage values of the modified core material with curing age.

The drying shrinkage values of CC and other specimens were tested, and the curing ages were from 1 day to 120 days (9 age stages). Correspondingly, the changing trend of CC was used as the reference baseline to evaluate the improvement degree of the fiber for dry shrinkage, and the relationship between the change rate of dry shrinkage and curing time was drawn, as shown in Figure 6.

It can be seen from the figure that the dry shrinkage value increases with curing age, and the curve of CC is in the middle of the overall ranges for all cases. The results indicate that the addition of fibers has a significant effect on dry shrinkage. In particular, the dry shrinkage values of C1205, C0615, C0910, and G1215 were improved significantly. Correspondingly, CF has a greater influence on dry shrinkage than GF and PF.

**Figure 7:**
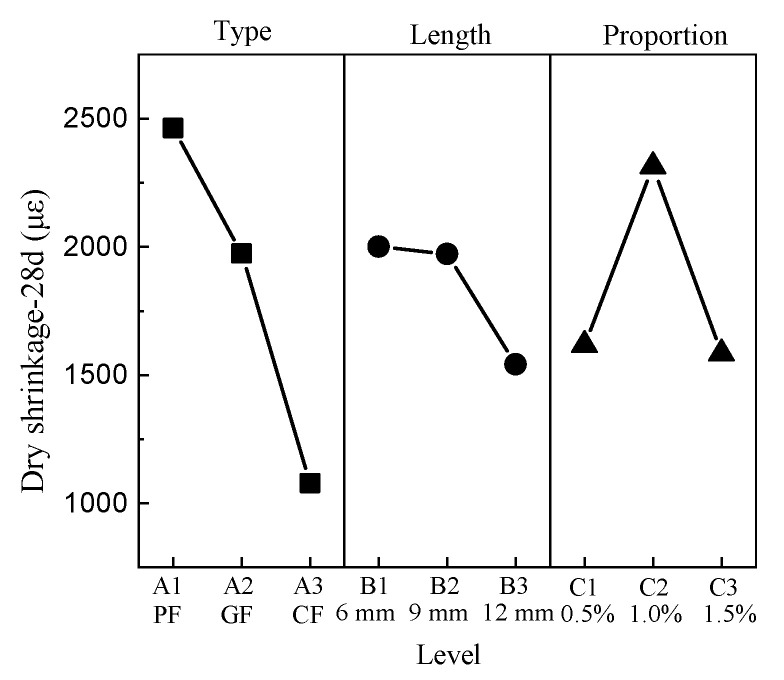
Mean dry shrinkage response.

The mean response of dry shrinkage at the standard age (28 days) is shown in Figure 7. For the type of fiber, the minimum and maximum values appear at level 3 and level 1, respectively. The dry shrinkage value has a negative correlation with the length factor; namely, the minimum value is at level 3 and the maximum value is at level 1. The minimum value of the proportion factor is level 3, and the maximum value is level 2.

### 3.6. Compressive Strength

**Figure 8:**
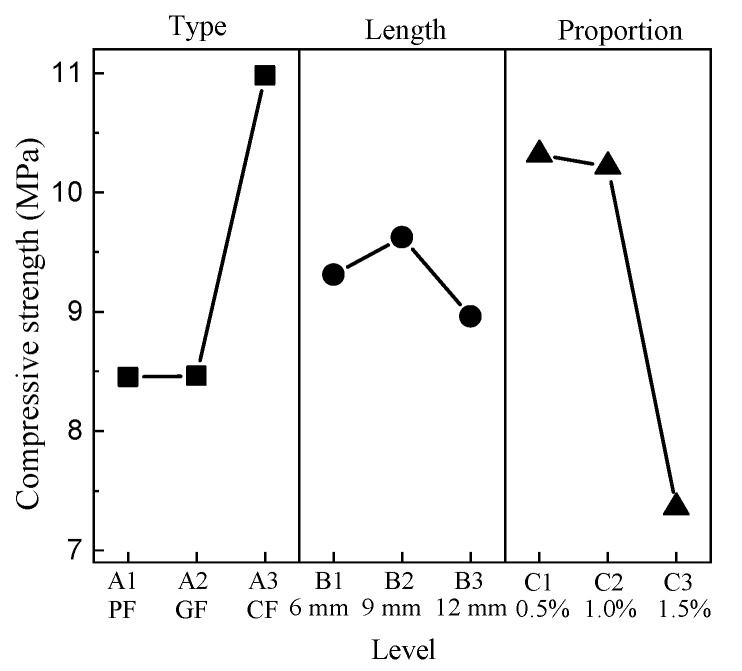
Mean compressive strength response.

The compressive strength ranges from 6.12 MPa to 12.35 MPa. G1215 has the minimum value, C1205 has the maximum value, and the compressive strength of CC is 8.58 MPa. The results show that the addition of fibers has an apparent effect on compressive strength. The mean response analysis of the compressive strength is shown in Figure 8.

For the type of fiber factor, the minimum value is level 1, and the maximum value is level 3. For the length factor, the minimum value appears at level 3, and the maximum value appears at level 2. There is a negative correlation between the compressive strength and the proportion of fibers, where the minimum is at level 3 and the maximum is at level 1.

### 3.7. Splitting Tensile Strength

**Figure 9:**
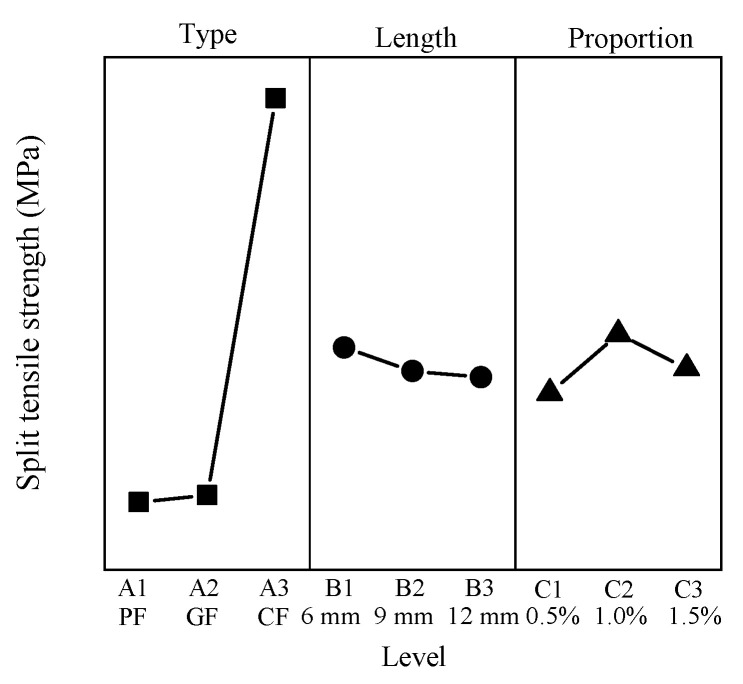
Mean splitting tensile strength response.

The splitting tensile strength ranges from 0.88 MPa to 2.35 MPa. P0605 has the minimum value, C0615 has the maximum value, and the splitting tensile strength of CC is 0.91 MPa. The results show that the addition of fibers has a great influence on the splitting tensile strength. The mean response analysis of the splitting tensile strength is shown in Figure 9.

The minimum value of the type factor appears at level 1, and the maximum value appears at level 3. The fiber length factor is negatively correlated with the fiber length factor, where the minimum appears at level 3 and the maximum appears at level 1. For the proportion factor, the minimum value appears at level 1, and the maximum value appears at level 2.

### 3.8. Uniaxial Compressive Strength

**Figure 10:**
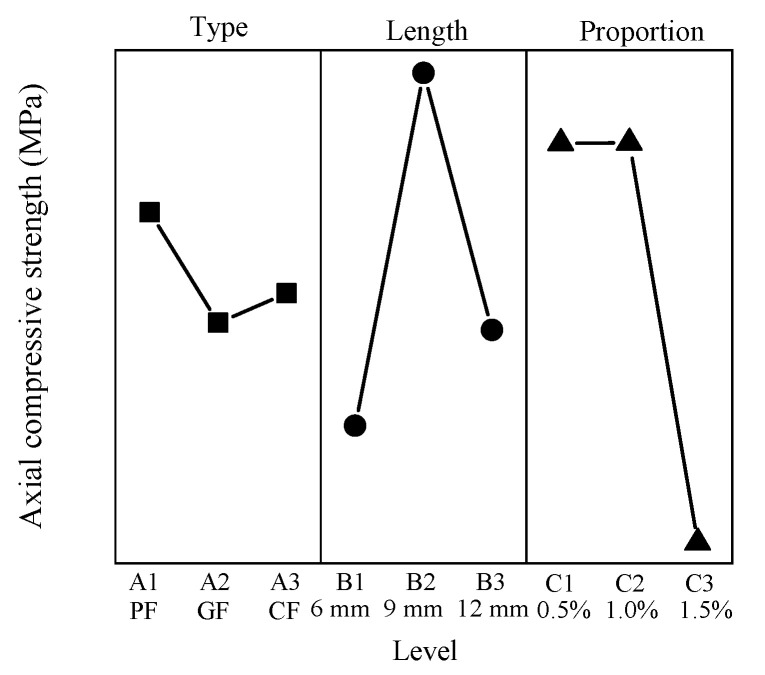
Mean axial compressive strength response.

The uniaxial compressive strength ranges from 3.24 MPa to 11.11 MPa, G1215 has the minimum value, C1205 has the maximum value, and the axial compressive strength of CC is 7.68 The results show that the addition of fibers significantly influences the axial compressive strength. The mean response analysis of the uniaxial compressive strength is shown in Figure 10.

For the axial compressive strength, the minimum value of the type factor appears at level 2, and the maximum value appears at level 1. The minimum value of the length factor appears at level 1, and the maximum value appears at level 2. The uniaxial compressive strength is negatively correlated with the proportion, where the minimum value appears at level 3 and the maximum value appears at level 1.

### 3.9. Static Elastic Modulus

**Figure 11:**
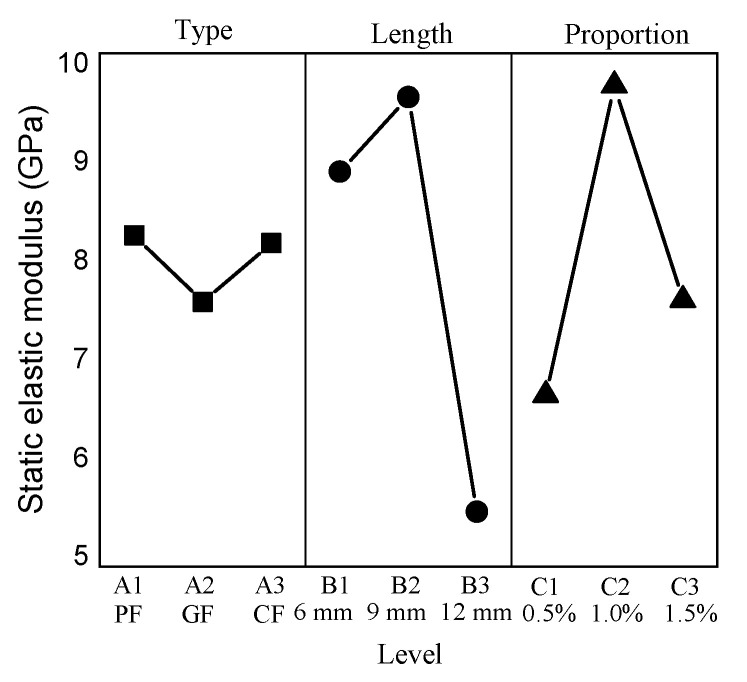
Mean static elastic modulus response.

The static elastic modulus ranges from 3.92 GPa to 11.32 GPa. C1205 has the minimum value, C0910 has the maximum value, and the static elastic modulus of CC is 6.79 GPa. The results show that the addition of fibers has an obvious effect on the static elastic modulus. The mean response analysis is shown in Figure 11.

The minimum value of the type factor appears at level 2, and the maximum value appears at level 1. The minimum value of the length factor appears at level 3, and the maximum value appears at level 1. For the proportion factor, the minimum value appears at level 1, and the maximum value appears at level 2.

### 3.10. Scanning Electron Microscope

**Figure 12:**
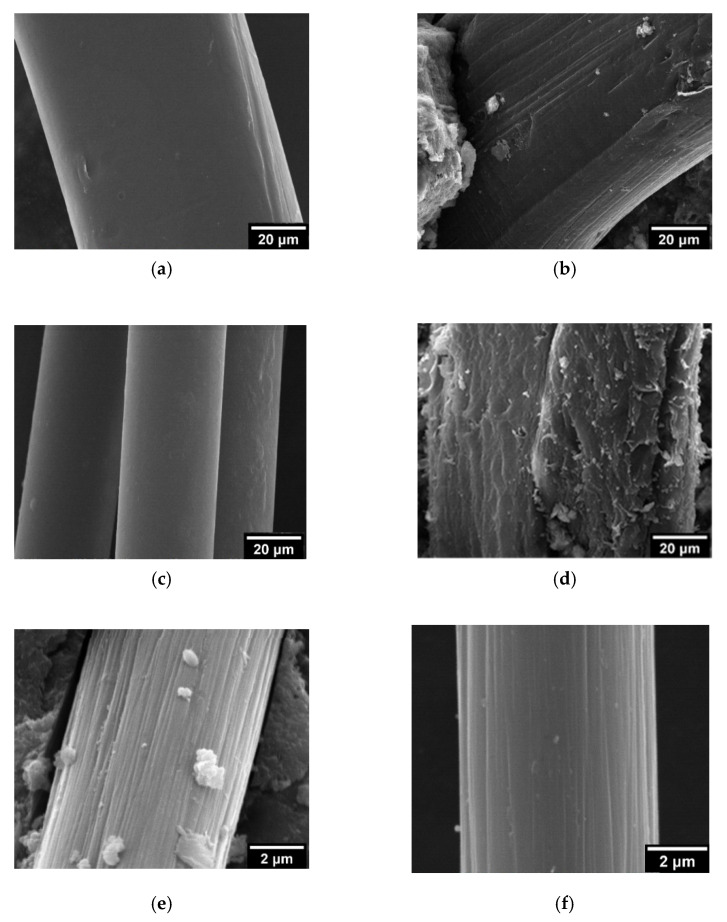
Micrographs of fiber surfaces: (**a**) original surface of PF; (**b**) surface of PF mixed in P0605; (**c**) original surface of GF; (**d**) surface of GF mixed in G0905; (**e**) original surface of CF; (**f**) surface of CF mixed in C1205.

The situation of the surface may be alterative during the mixing and forming processes of concrete. Hence, a rougher fiber surface is favorable for combining the concrete matrix and fibers [44]. Figure 12a,c,e show the apparent morphology of the fiber before being mixed into EPS concrete, and Figure 12b,d,f show the surfaces of the fibers added into EPS concrete.

As shown in Figure 12a,b, the surface of PF is quite smooth, but its surface presents relatively regular vertical microcracks after being mixed in concrete.

Although a small number of hydration crystals is visibly attached to its surface, it looks smoother compared with the surface of GF and CF in EPS concrete. Therefore, the boundary between PF and concrete is inferior to that of the others, which accounts for the general lower mechanical properties of EPS concrete mixed with PF.

As shown in Figure 12c,d, a relatively smooth GF surface was observed. However, the surface of GF became extremely rough, and lamellar spalling damage appeared, resembling fish scales after it was mixed in EPS concrete. This probably accounts for the alkaline environment of the cement matrix and the chemical etching caused by Ca(OH)_2_ [45,46]. However, the mechanical properties of GF are weakened by damage to its surface. The roughness of the surface is conducive to the attachment of massively hydrated crystals and the synergistic bonding between fibers and the cement matrix. The mechanical properties of EPS concrete mixed with GF are generally better than those of concrete mixed with PF, but the overall improvement is not obvious.

As shown in Figure 12e,f, some regular fine strips on the surface of CF emerged. The diameter and contact area with the cement matrix of CF are smaller and larger, respectively than those of PF and GF. The formation and adhesion of the hydration crystals are promoted by the increase in the fine strips on the surface of CF after it is mixed in concrete. Furthermore, it can be observed that massive crystals are attached to the surface of CF, making it more efficient for the bonding and adhesion of hydration crystals. Moreover, the mechanical properties of EPS concrete are favorably improved, which is confirmed by the compressive strength and splitting tensile strength results in the OED.

## 4. Application

Commercial sandwich panels (CSPs) are a common type of prefabricated concrete panels that commonly use EPS concrete as their core material. The tensile strength of EPS concrete becomes the controlling factor [47,48,49] for the safety guarantee of sandwich panels when they encounter moments of flexure. Hence, as the core material of CSPs, the optimal combination (OC) of EPS concrete was selected according to the splitting tensile strength.

**Figure 13:**
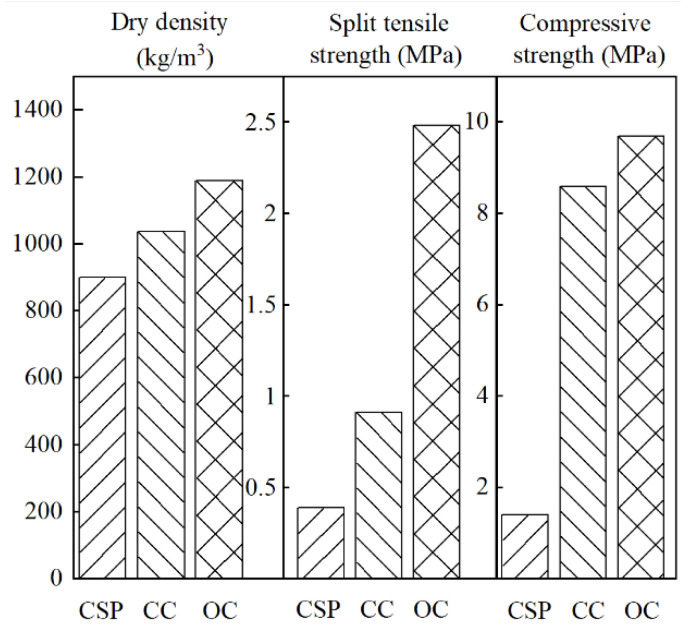
Comparison of properties.

In addition, other performance tests were performed on OC and compared with CC and the CSP core [50] material. The results are shown in Figure 13. It can be seen from the test results that the dry density, splitting tensile strength, and compressive strength of OC compared to the CSP material increased by 31%, 591%, and 536%, respectively. For CC, the dry density, splitting tensile strength, and compressive strength are higher than those of OC and are 14%, 172%, and 13%, respectively.

Hence, the mechanical properties of CSPs are predominantly improved after modification by adding fibers. The density of the core material is increased by approximately 30%, but there is a 600% increase in the mechanical properties.

Tests of dry density, compressive strength, and splitting tensile strength under OC at different curing ages and the influence of age on its properties were obtained. The age stages of this test were 7, 14, 28, 56, and 90 days. Three samples of each age stage were tested to obtain the dry density, compressive strength, and splitting tensile strength. The average value of each test result is shown in Table 8.

### 4.1. Dry Density

**Figure 14:**
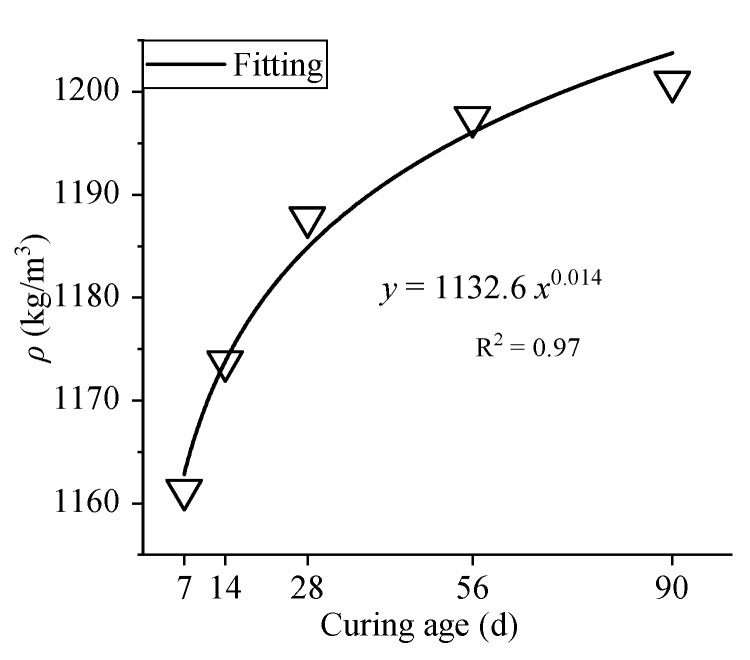
Curve fitting of dry density.

As shown in Table 8, the dry density of OC at different ages ranges from 1161.28 kg/m^3^ to 1200.95 kg/m^3^. The curve fitting of the dry density is shown in Figure 14.

The dry density continues to improve with increasing curing time, and the rate of increase gradually slows down as the age increases. When the age increases from 7 days to 28 days, 14 days to 28 days, 28 days to 56 days, and 28 days to 90 days, the dry density increases by 1%, 2%, 0.8%, and 1%, respectively. Although increasing age has an impact on the dry density, the improvement is not significant compared to that at the standard age; the improvement is only approximately 1% to 2%.

### 4.2. Compressive Strength

**Figure 15:**
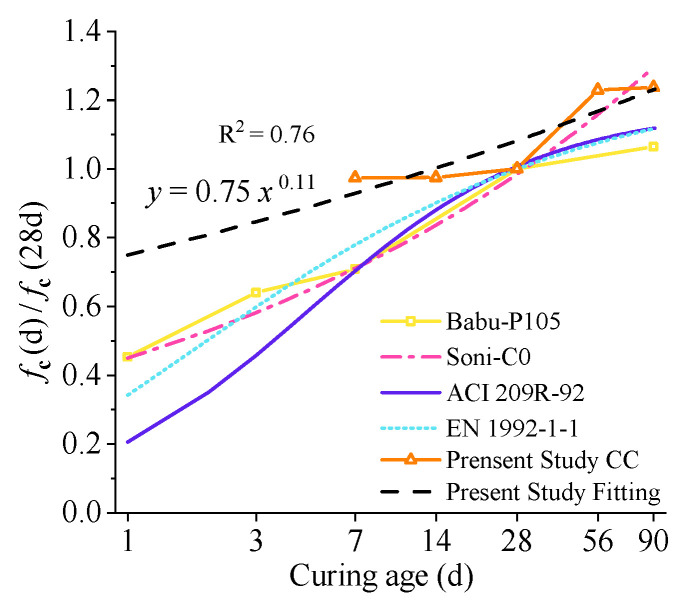
Curve fitting of the compressive strength.

The compressive strength of OC at different ages ranges from 9.43 MPa to 11.98 MPa. The curve fitting of dry density is shown in Figure 15.

The compressive strength continues to improve with increasing curing time, and the rate of increase gradually slows down as the age increases. When the age increases from 7 days to 28 days, 14 days to 28 days, 28 days to 56 days, and 28 days to 90 days, the dry density increases by 2.5%, 2.5%, 23%, and 24%, respectively. It shows that the compressive strength of OC at an early age is approximately 98% of that at the standard age and is higher than the compressive strength of CC at the standard age. The compressive properties of OC increase significantly after reaching the standard age, but the growth rate slows down after 56 days. As shown in Figure 15, compared with the previous study [51,52] and the prediction curve of ordinary concrete [53,54], it was found that the compressive strength increased with increasing age. At an early age (within 14 days), the compressive strength of OC concrete is markedly superior to that of ordinary concrete. For instance, compared with P105, the compressive strength of OC at 7 days increased by nearly 110%. The ITZs between CF and the cement matrix significantly improved at the early age stage due to the high hydration degree, which enhanced the mechanical properties of the concrete material.

### 4.3. Splitting Tensile Strength

**Figure 16:**
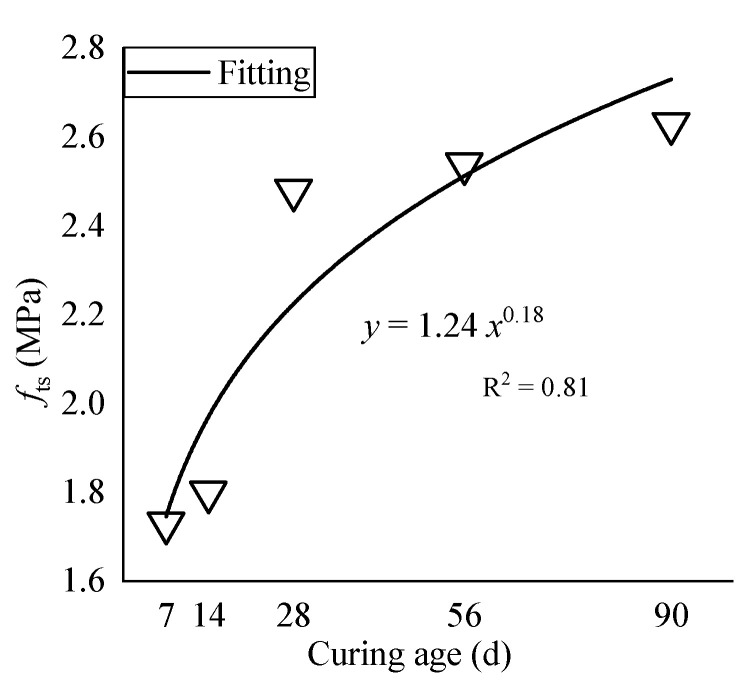
Curve fitting of the splitting tensile strength.

The splitting tensile strength of OC at different ages ranges from 1.73 MPa to 2.63 MPa. The fitting curve of the dry density is shown in Figure 16.

When the age increases from 7 days to 28 days and from 14 days to 28 days, the splitting tensile strength increases by 0.75 MPa and 0.68 MPa, namely, 1% and 2%, respectively. When the age increases from 28 days to 56 days and 90 days, respectively, the tensile strength increases by 0.06 MPa and 0.15 MPa, which are improvements of 2% and 6%, respectively. However, after reaching the standard age, the splitting tensile strength continues to increase, but the improvement is not substantial. The splitting tensile strength continues to improve with increasing curing time, and the rate of increase gradually slows down as the age increases. When the age increases from 7 days to 28 days, 14 days to 28 days, 28 days to 56 days, and 28 days to 90 days, the dry density increases by 1%, 2%, 2%, and 6%, respectively. After reaching the standard age, the splitting tensile strength continues to increase, but the rate of increase is not sharp.

### 4.4. Scanning Electron Microscope

**Figure 17:**
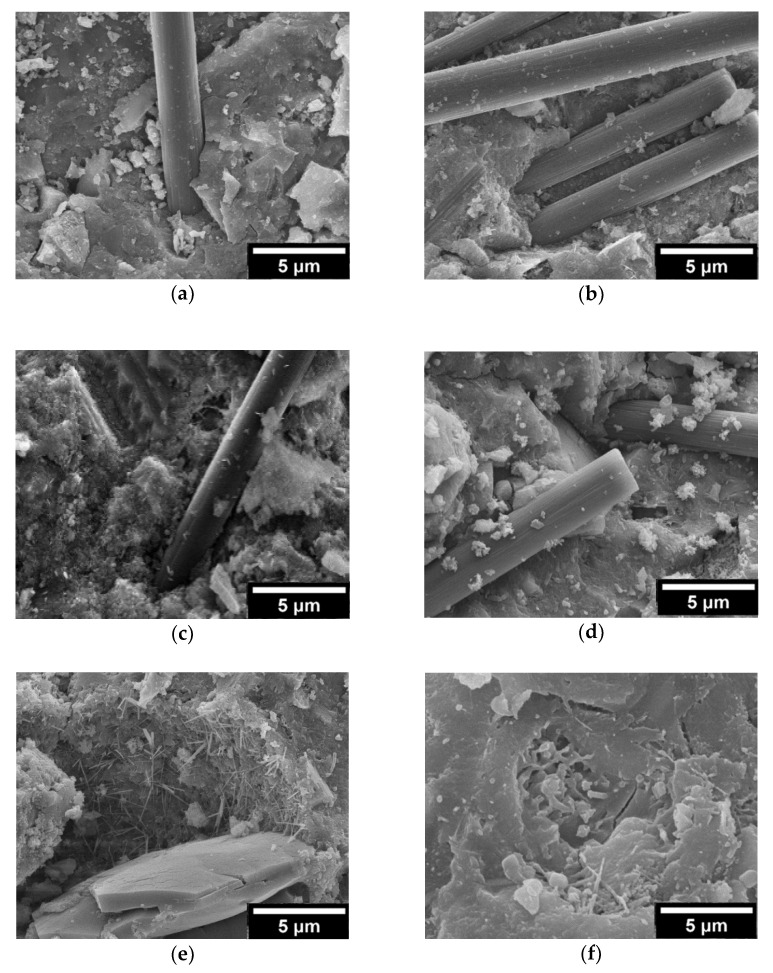

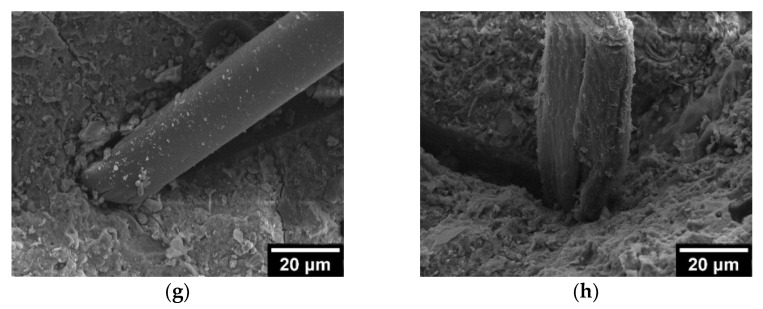
Microscopic morphological analysis: (**a**) OC in 7 days; (**b**) OC in 14 days; (**c**) OC in 28 days; (**d**) OC in 120 days; (**e**) Aft in 120 days; (**f**) Aft in 120 days; (**g**) P0605 in 28 days; (**h**) G0905 in 28 days.

The OC material at four ages (7, 14, 28, and 120 days) was analyzed by SEM. As shown in Figure 17a–d, there is no obvious gap near the ITZ between the cement matrix and the roots of CF at all ages. The ITZs at the roots of PF and GF are shown in Figure 17g,h and are more apparent than those in CF. Compared with the curing conditions at the standard age, the bonding strength between the fiber and matrix is already tight at an early age, which explains why the mechanical properties of OC at an early age are significantly higher than those of CC at a standard age.

Clustered and thickened hydrated crystals are attached to the matrix and fibers of the surface and increase with age. Moreover, the overall mechanical properties are further enhanced, which may be attributed to the large number of hydration crystals observed on the surface of CF in the SEM micrographs, making the connection between the fiber root and the cement matrix closer.

As shown in Figure 17e,f, partial micropores attached to denser acicular ettringite hydrated crystals are visible in the cement matrix at a prolonged age (120 days) and account for the enhancement of the mechanical properties.

### 4.5. X-ray Diffraction

**Figure 18:**
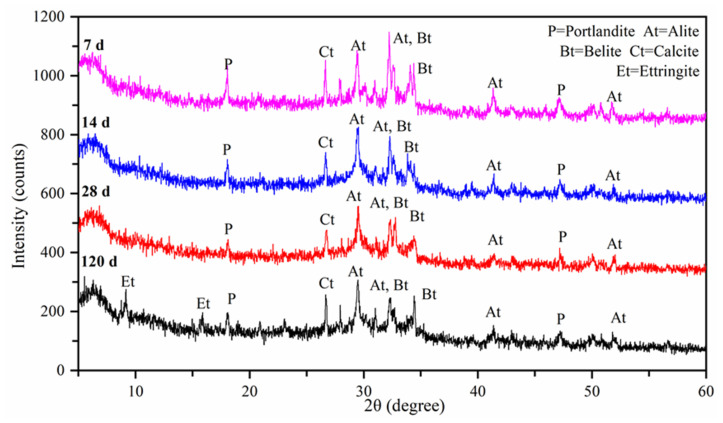
XRD diffraction pattern.

The OC material at ages 7, 14, 28, and 120 days was characterized by XRD, as shown in Figure 18.

The various phases of the different ages are roughly the same. The main phases are the calcite and portlandite phases, as well as the nonahydrate alite and belite phases.

Similarly, the diffraction peaks of alite and belite (Ca_3_SiO_3_ and Ca_2_SiO_4_) at 7 days and 14 days are more significant than those at 28 days, especially the peaks at 7 days. The above shows that the longer the curing period is, the more sufficient the hydration degree is SiO_2_ continuously reacts with the cement matrix for secondary hydration, which is the main component of SF mixed in concrete. Ca(OH)_2_ is consumed continuously, which sufficiently promotes the secondary hydration of OC as the curing age increases. Accordingly, this is corroborated by the phenomenon observed by SEM.

The diffraction peak of Aft, namely, ettringite, was observed in the XRD pattern at the age of 120 days, accounting for the long curing period and the greater the degree of hydration. In fact, the adhesion between the fibers and the cement matrix is also improved when the hydration crystals are increased. Consequently, the mechanical properties of concrete are more effectively improved by the above two effects, hydration, and adhesion, whose effects are amplified when they are used together.

## 5. Conclusions

To investigate the effect of fibers on EPS concrete under the factors of the fiber type, length, and proportion, an OED with three factors and three levels was designed. The material, mechanical properties, and microstructure of EPS concrete with fibers were tested. Furthermore, based on the CSP in the practice project, taking the splitting tensile strength as the selection criterion, OC was predicted. The dry density, the splitting tensile strength, the compressive strength, SEM, and XRD of the specimens at various ages were analyzed, and the following conclusions were obtained.

The addition of fibers reduced the fluidity and workability of EPS concrete to a great extent. For the dry density, water absorption rate, and UPV, the addition of fibers had little effect. However, the dry shrinkage rate was considerably altered after the fibers were mixed, and the overall modification effect of CF was better than that of PF and GF. The compressive strength, splitting tensile strength, uniaxial compressive strength, and static elastic modulus of EPS concrete with fibers were significantly improved. Among them, the improvement in CF was the most remarkable. The microscopic analysis showed that the mixing of fibers promotes the hydration of concrete. Correspondingly, with increasing curing time, the hydration effect is also better. Furthermore, the hydration crystals considerably enhance the connection between the fibers and cement matrix, thereby improving the overall mechanical properties of the concrete. CF has the most marked improvement in mechanical properties due to its large contact area with the cement matrix and the large number of hydration crystals attached to its surface.

Compared with CC, the dry density, splitting tensile strength, and compressive strength of OC increased by 14%, 172%, and 13%, respectively. Similarly, compared with the CSP, the dry density, splitting tensile strength, and compressive strength of OC increased by 31%, 591%, and 536%, respectively. That is, the mechanical properties are notably enhanced. The compressive strength increased rapidly at an early age and reached 98% of the compressive strength at the standard age. There was no obvious gap near the ITZ between CF and the cement matrix at all ages, which was observed by SEM and XRD analyses. Moreover, with increasing curing age, the abundant hydrated crystals on the surface of CF also multiplied, which is conducive to the further enhancement of mechanical properties. Moreover, the appearance of ettringite was observed at 120 days, which also proved that the addition of fibers can promote the hydration of concrete.

## Figures and Tables

**Table 1:** Chemical composition and performance indicators.

SO_3_	MgO	Cl^−^	Slag	Gypsum	Ignition Loss	Specific Surface Area
2.20%	3%	0.04%	12%	6%	4%	358 m^2^/kg

**Table 2:** Chemical composition of silica fume.

Composition	SiO_2_	Al_2_O_3_	Fe_2_O_3_	MgO	CaO	Na_2_O
w/%	96.74%	0.32	0.008	0.1	0.11	0.09

**Table 3:** River sand sieving analysis.

Sieve Hole (mm)	9.5	4.75	2.36	1.18	0.6	0.3	0.15	<0.15
sand sieve weight (g)	691.0	705.0	681.2	572.0	561.2	554.0	555.0	464.8
record weight (g)	691.0	705.0	688.4	625.8	643.2	673.0	684.4	572.0
sand sieve allowance (g)	0.0	0.2	7.2	53.8	82.0	19.0	129.4	107.2
sieve allowance percentage (%)	0.0	0.0	1.4	10.8	16.5	23.9	25.9	21.5
cumulative sieve remainder percentage (%)	0.0	0.0	1.0	12.0	29.0	53.0	79.0	100.0
through percentage	100.0	100.0	99.0	88.0	71.0	47.0	21.00	0.00
fineness modulus	1.73							

**Table 4:** Physical properties.

Type	Density	Tensile Strength	Elastic Modulus	Breaking Elongation
PF	0.91 g/cm^3^	360 MPa	4236 MPa	28.4%
GF	2.5 g/cm^3^	469 MPa	4286 MPa	21.6%
CF	1.80 g/cm^3^	4900 MPa	240,000 MPa	2.1%

**Table 5:** OED table.

Level	Factor		
	A-Type	B-Length (mm)	C-Proportion (%)
1	PF	6	0.5
2	GF	9	1.0
3	CF	12	1.5

**Table 6:** Mix design.

Mix Designation	Cement (kg/m^3^)	SF (kg/m^3)^	Water (kg/m^3^)	NRS (kg/m^3^)	SP (kg/m^3^)	EPS (kg/m^3^)	Fiber		
							Type	Length (mm)	Proportion (kg/m^3^)
CC	508.91	127.23	152.67	407.12	4.07	10	-	-	-
P0605	508.91	127.23	152.67	407.12	4.07	10	PF(A1)	6 (B1)	4.55 (C1)
G0905	508.91	127.23	152.67	407.12	4.07	10	GF(A2)	9 (B2)	12.50(C1)
C1205	508.91	127.23	152.67	407.12	4.07	10	CF(A3)	12(B3)	9.10 (C1)
C0910	508.91	127.23	152.67	407.12	4.07	10	CF(A3)	9 (B2)	18.20(C2)
G0610	508.91	127.23	152.67	407.12	4.07	10	GF(A2)	6 (B1)	25.00(C2)
P1210	508.91	127.23	152.67	407.12	4.07	10	PF(A1)	12(B3)	9.10 (C2)
P0915	508.91	127.23	152.67	407.12	4.07	10	PF(A1)	9 (B2)	13.65(C3)
G1215	508.91	127.23	152.67	407.12	4.07	10	GF(A2)	12(B3)	37.50(C3)
C0615	508.91	127.23	152.67	407.12	4.07	10	CF(A3)	6 (B1)	27.30(C3)

**Table 7:** Material and mechanical properties.

Mix Designation	Slump (mm)	Dry Density (kg/m^3^)	Water Absorption (%)	UPV (km/s)	Compressive Strength (MPa)	Splitting Tensile Strength (MPa)	Uniaxial Compressive Strength (MPa)	Static Elastic Modulus (GPa)
CC	196	1036.60	5.5	2.86	8.58	0.91	7.68	6.97
P0605	115	1043.71	6.1	2.83	9.41	0.88	6.63	7.51
G0905	10	1083.38	3.5	3.02	9.19	1.17	9.98	8.48
C1205	7	1045.44	5.4	2.91	12.35	2.04	11.11	3.92
C0910	1	1151.20	4.5	3.02	12.16	2.04	8.86	11.32
G0610	12	1069.16	4.4	3.01	10.08	1.23	10.09	9.91
P1210	72	1118.93	4.4	2.90	8.41	1.30	8.78	8.09
P0915	11	986.42	5.9	2.74	7.53	1.06	10.61	9.17
G1215	2	1085.07	9.5	3.00	6.12	0.89	3.24	4.35
C0615	1	1097.71	5.7	2.92	8.43	2.35	4.06	9.29

**Table 8:** Effect of age (7 to 90 days) on OC.

Name	Age (days)	Dry Shrinkage (kg/m^3^)	Compressive Strength (MPa)	Splitting Tensile Strength (MPa)
OC-7 d	7	1161.28	9.43	1.73
OC-14 d	14	1173.80	9.44	1.80
OC-28 d	28	1187.78	9.68	2.48
OC-56 d	56	1197.55	11.90	2.54
OC-90 d	90	1200.95	11.98	2.63

## Data Availability

The data presented in this study are available on request from the corresponding author.

## Abbreviations

EPS	Expanded polystyrene
FA	Fly ash
SF	Silica fume
ITZ	Interfacial transition zone
OPC	Ordinary Portland cement
NRS	Natural river sand
PF	Polypropylene fiber
GF	Glass fiber
CF	Carbon fiber
SP	Superplasticizer
CC	Control case
OED	Orthogonal experimental design
UPV	Ultrasonic pulse velocity
SEM	Scanning electron microscope
XRD	X-ray diffraction
CSP	Commercial sandwich panel
OC	Optimal combination
